# Supplementary material for: Known Drugs Identified by Structure-Based Virtual Screening Are Able to Bind Sigma-1 Receptor and Increase Growth of Huntington Disease Patient-Derived Cells
Source: Int J Mol Sci. 2021 Jan 28;22(3):1293. doi: 10.3390/ijms22031293 (PMC7865886; doi:10.3390/ijms22031293)
Supplement: Supplementary file 1 [file ijms-22-01293-s001.pdf]

Supplementary Information

Table S1. All-against-all structure comparison of σ1R ligand binding domain in the available 3D structures determined by X-ray crystallography. The ligand binding domain comprises residues 29-212 in 5HK2 chain B and 35-218 in all other chains. RMSD values calculated after optimal all-against-all pairwise superposition of either Cα or all atoms are reported in the lower and upper part of the matrix, respectively.

		5HK1			5HK2			6DJZ			6DK0			6DK1		
		A	B	C	A	B	C	A	B	C	A	B	C	A	B	C
5HK1	A	-	0.34	0.45	0.37	0.44	0.28	0.14	0.36	0.46	0.17	0.37	0.47	0.38	0.58	0.53
	B	0.66	-	0.31	0.31	0.33	0.36	0.35	0.14	0.34	0.37	0.18	0.35	0.49	0.44	0.44
	C	0.77	0.60	-	0.48	0.19	0.49	0.46	0.34	0.14	0.49	0.37	0.19	0.60	0.51	0.42
5HK2	A	0.73	1.23	0.79	-	0.46	0.33	0.37	0.33	0.51	0.39	0.34	0.52	0.50	0.55	0.54
	B	0.76	0.61	0.49	0.79	-	0.47	0.44	0.35	0.20	0.47	0.37	0.20	0.55	0.50	0.40
	C	0.59	0.69	0.66	0.70	0.81	-	0.28	0.37	0.51	0.28	0.38	0.52	0.44	0.58	0.56
6DJZ	A	0.18	0.67	0.78	0.70	0.77	0.60	-	0.35	0.46	0.11	0.36	0.46	0.35	0.57	0.52
	B	0.66	0.18	0.62	0.65	0.62	0.68	0.66	-	0.34	0.37	0.11	0.35	0.48	0.41	0.43
	C	0.72	0.62	0.19	0.60	0.50	0.78	0.72	0.62	-	0.49	0.36	0.14	0.58	0.50	0.39
6DK0	A	0.26	0.69	0.26	0.75	0.80	0.61	0.22	0.67	0.75	-	0.37	0.49	0.33	0.56	0.52
	B	0.66	0.22	0.64	0.66	0.65	0.70	0.67	0.16	0.64	0.68	-	0.37	0.48	0.40	0.43
	C	0.73	0.62	0.24	0.83	0.50	0.80	0.73	0.63	0.17	0.75	0.64	-	0.56	0.46	0.36
6DK1	A	0.55	0.82	0.92	0.83	0.88	0.75	0.53	0.80	0.87	0.52	0.80	0.86	-	0.53	0.47
	B	0.93	0.67	0.83	0.84	0.80	0.98	0.92	0.61	0.79	0.93	0.63	0.77	0.83	-	0.40
	C	0.89	0.79	0.61	0.90	0.71	0.91	0.89	0.78	0.57	0.89	0.78	0.55	0.81	0.79	-

Supplementary Figure 1. Ligand binding to σ1R. The protein backbone in co-ordinate file 5HK1 is shown as transparent green ribbon. σ1R residues involved in ligand binding in the 3D structures listed in Table 1 are labelled, shown as sticks and colored by atom type: C, green; N, blue; O, red. Ligands are shown as sticks and colored by atom-type: C, white; N, blue; O, red; S, yellow F, light green. Glu172 side-chain oxygen and the closest ligand atom able to establish a polar interaction with it are connected by a dashed line. The co-ordinates of the complex with PD144418 **(a)** have been experimentally determined by X-ray crystallography (PDB ID: 5HK1). The conformation of Pridopidine **(b)** was predicted by virtual screening. The conformation of all the other ligands corresponds to the best energy pose of the most populated cluster predicted by docking simulations (see Materials and Methods): **(c)** Iloperidone; **(d)** Paliperidone; **(e)** Nilotinib; **(f)** Vilazodone; **(g)** Linagliptin; **(h)** Flibanserin.

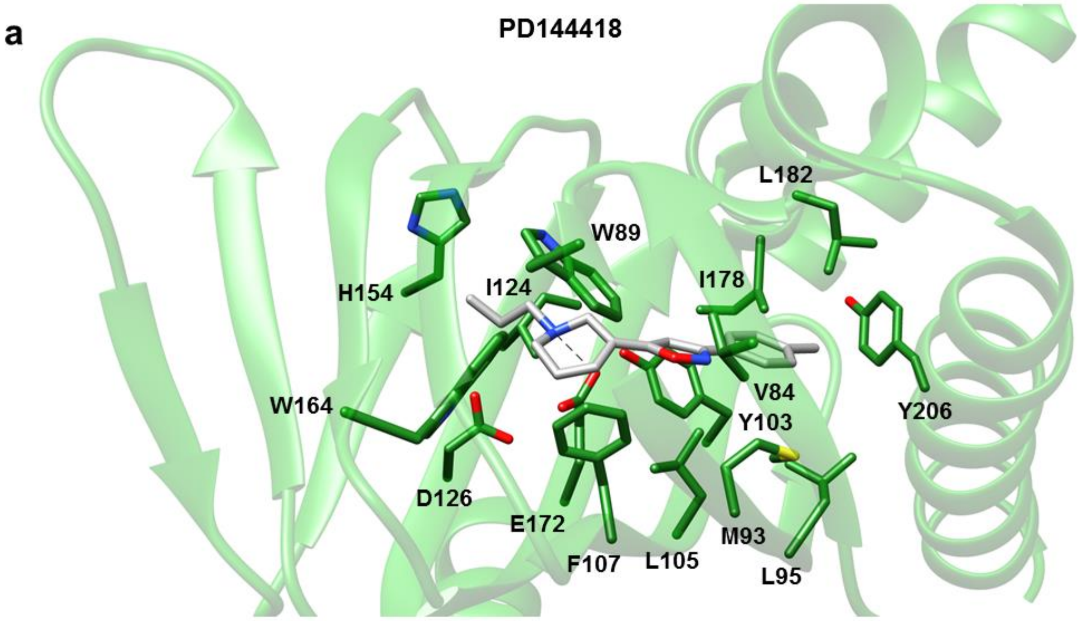
a
PD144418
L182
W89
I178
H154
I124
Y206
V84
W164
Y103
D126
E172
M93
F107
L105
L95

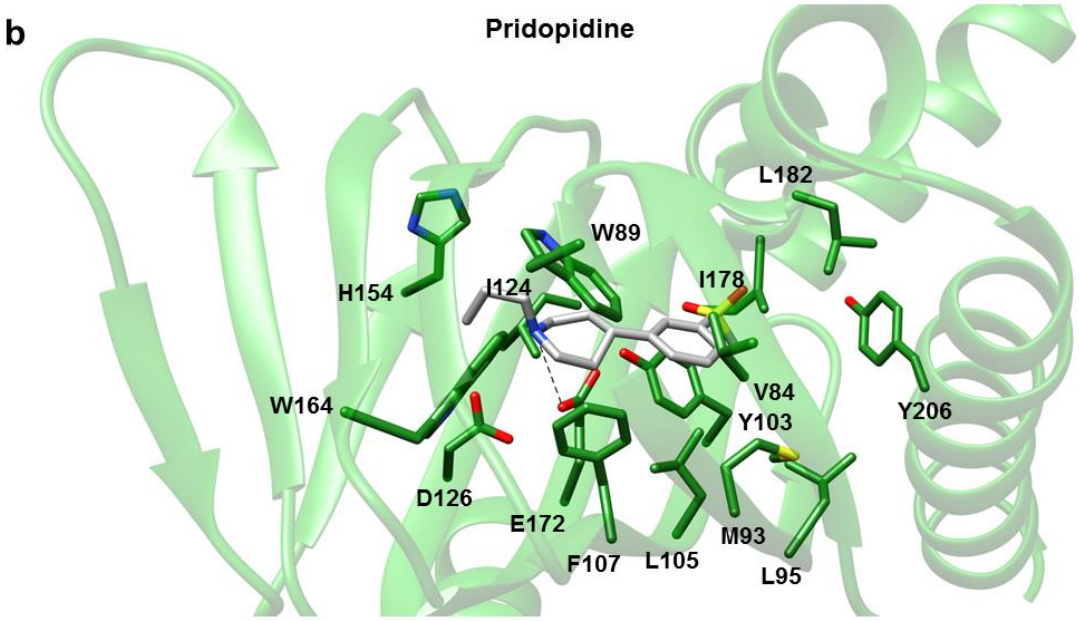
b
Pridopidine
L182
W89
I178
H154
I124
Y206
V84
W164
Y103
D126
E172
M93
F107
L105
L95

c

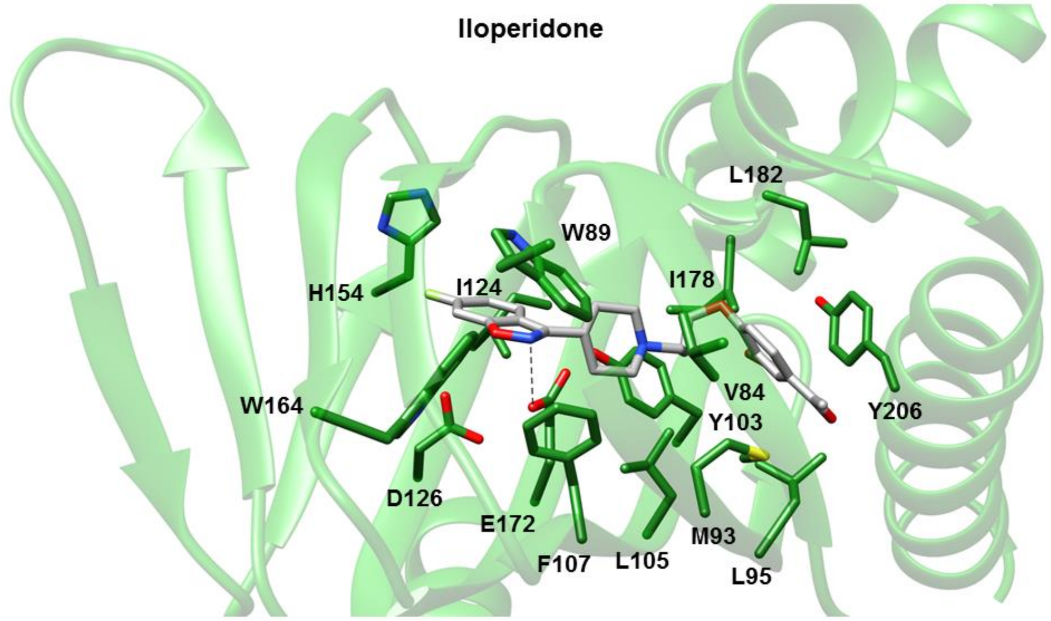
Iloperidone
L182
W89
I178
H154
I124
V84
W164
Y103
Y206
D126
E172
M93
F107
L105
L95

d

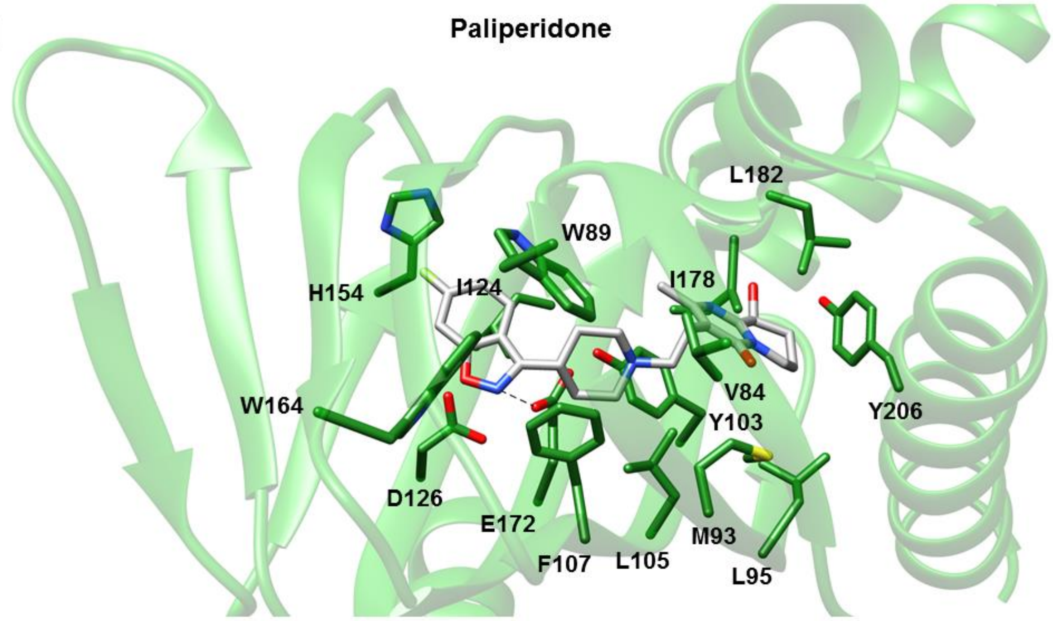
Paliperidone
L182
W89
I178
H154
I124
V84
W164
Y103
Y206
D126
E172
M93
F107
L105
L95

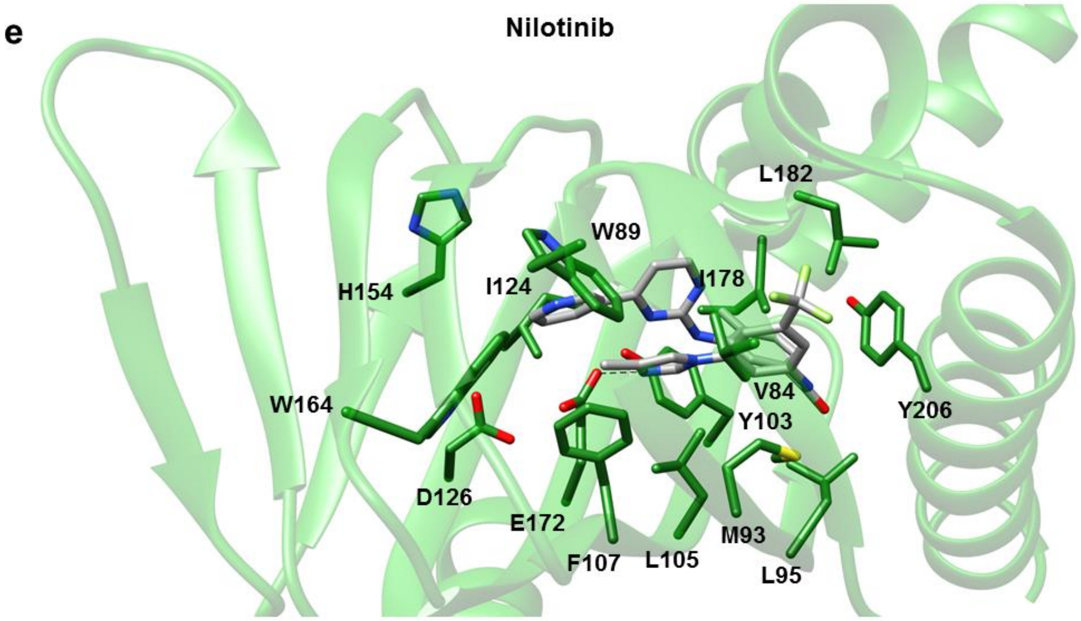
e
Nilotinib
L182
W89
I178
H154
I124
W164
V84
Y103
Y206
D126
E172
F107
L105
M93
L95

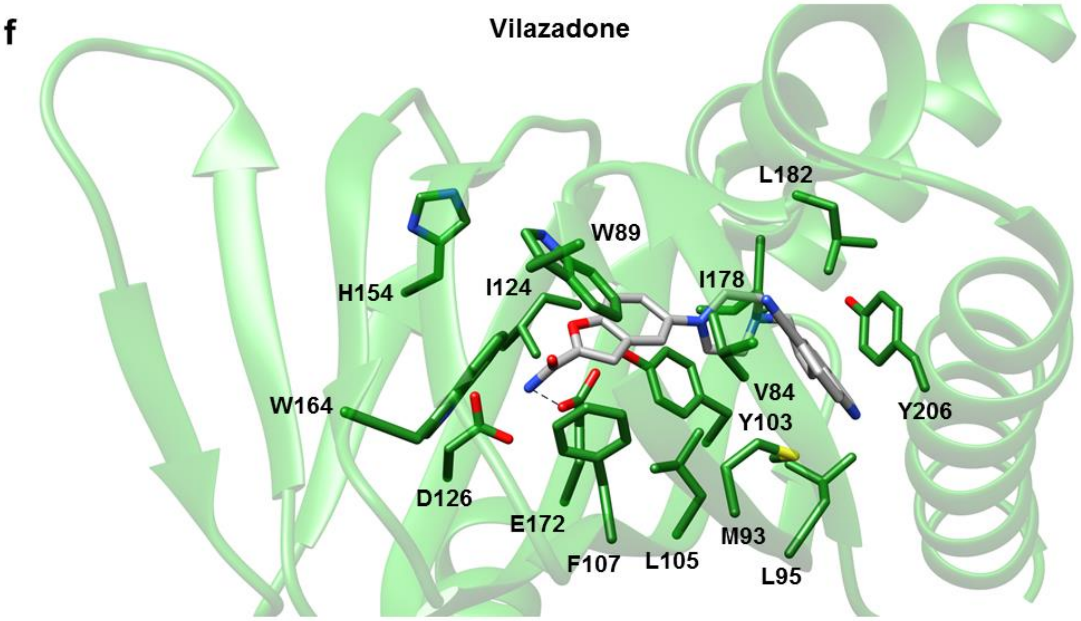
f
Vilazadone
L182
W89
I178
H154
I124
W164
V84
Y103
Y206
D126
E172
F107
L105
M93
L95

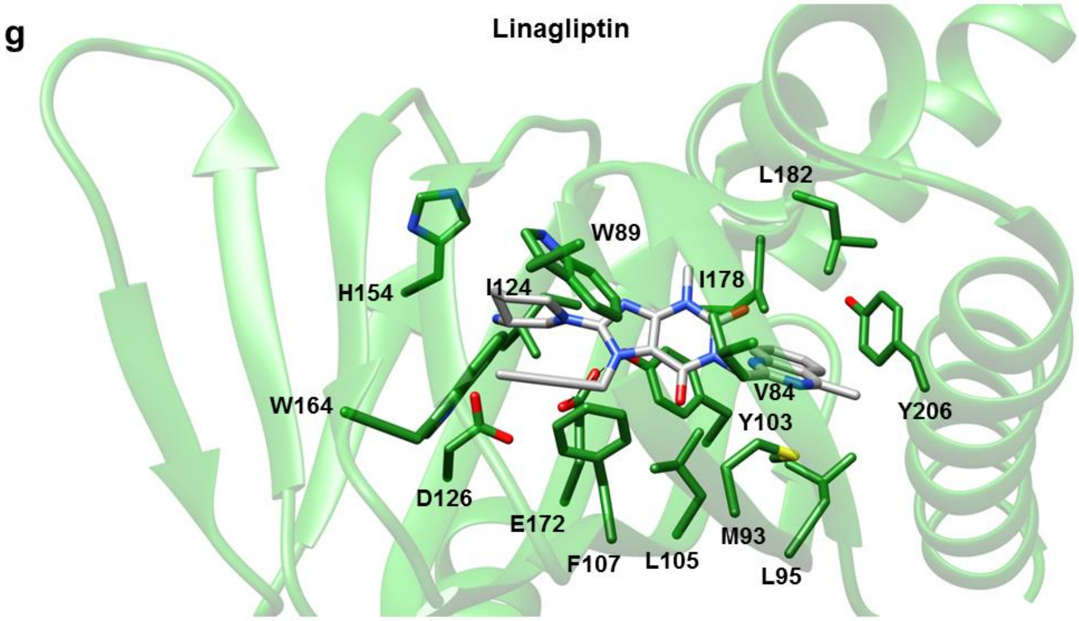

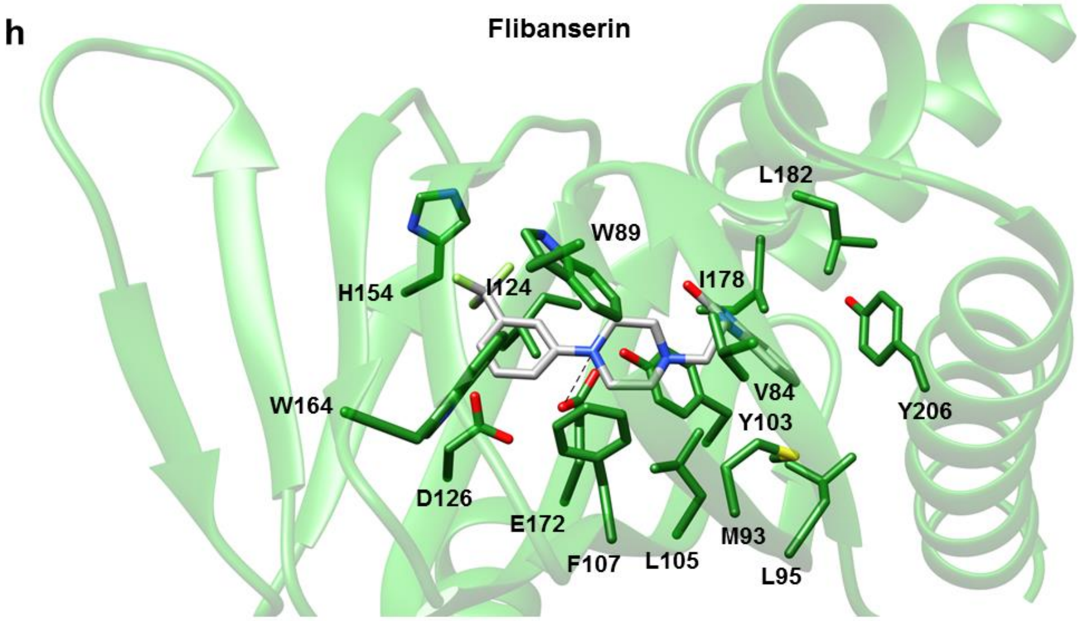

Figure S1. Ligand binding to σ1R title
